# Acute kidney failure following severe viper envenomation: clinical, biological and ultrasonographic aspects

**DOI:** 10.1590/1678-9199-JVATITD-2020-0059

**Published:** 2020-12-07

**Authors:** Blaise Adelin Tchaou, Kofi-Mensa Savi de Tové, Charles Frédéric Tchégnonsi N’Vènonfon, Patrick Kouomboua Mfin, Abdou-Rahman Aguemon, Martin Chobli, Jean-Philippe Chippaux

**Affiliations:** 1 Department of Anesthesia-Resuscitation and Emergency, Borgou-Alibori University Hospital Center, Parakou, Bénin.; 2 Department of Radiology and Medical Imaging, Borgou-Alibori University Hospital Center, Parakou, Bénin.; 3 University of Paris, MERIT, IRD, Paris, France.; 4 CRT, Pasteur Institute, Paris, France.

**Keywords:** Envenomation, Snakebite, Antivenom, Acute kidney injury, KDIGO, Subcapsular hematoma, Hemorrhagic syndrome, Benin

## Abstract

**Background:**

Acute kidney injury (AKI) is a frequent complication of snakebite envenomation, which is still little known in sub-Saharan Africa. This study aims to describe the clinical, biological and ultrasonographic aspects of AKI following severe snakebite envenomation managed in the intensive care unit.

**Method:**

A prospective observational survey was performed in Benin over a period of 18 months. All patients suffering severe snakebite envenomation (SBE) were included. The diagnosis of AKI was made using the KDIGO criteria. Kidney ultrasound exam was performed in all patients to assess internal bleeding and morphological and structural abnormalities of the kidneys.

**R**esults**:**

Fifty-one cases of severe SBE were included. All patients presented inflammatory syndrome and showed abnormal WBCT whereas bleeding was found in 46 of them (90%). The median time to hospital presentation was three days. The majority of patients were male (M/F sex ratio = 1.55) and the median age was 26. Sixteen patients (31%) showed AKI according to the KDIGO criteria. Severe AKI (KDIGO stage 2 and 3) was observed in three patients, including one stage 2 and two stage 3. Kidney ultrasound revealed three cases of kidney capsular hematoma (6%), two cases of kidney hypertrophy (3%), three cases of kidney injury (4%), two stage 1 KDIGO and one stage 2 KDIGO. Only one patient benefited from hemodialysis. All patients showing AKI recovered without sequels. The median duration of hospital stays was four days. Seven patients died (14%) including four among the 16 AKI patients. Antivenom has been administered to 41 patients (80%). The comparison between patients without and with AKI did not show any significant difference except gender (p = 10^-2^).

**C**onclusion**:**

AKI is a common complication of severe snakebite envenomation. Resulting from inflammatory and hemorrhagic disorders, AKI may prove to be a short-term life-threatening factor.

## Background

Severe snakebite envenomation is a medical and surgical emergency and a real public health issue in most tropical countries, particularly in low- and middle-income ones [1]. Every year, it is estimated that about 5 million snakebites occur, resulting in nearly 2.5 million severe envenomations, 150,000 deaths and up to 400,000 cases of disability of variable importance (amputations, blindness, major scars etc.) [2-4]. More than 95% of the cases of envenomation occurs in sub-Saharan Africa (SSA) and in South Asia [5,6].

In Benin, the annual snakebite incidence is around 400 per 100,000 inhabitants, and more than 4,000 patients require medical care [7-10]. Mortality is estimated at 10 deaths per 100,000 inhabitants based on household surveys in the rural areas of center and north Benin, i.e., more than 500 deaths per year. However, official figures by the health authorities are around only 100 deaths annually [9]. Health center case fatality rates are 1-10% higher in the north of the country due to the abundance of *Echis ocellatus*, a small Viperidae snake whose venom is highly inflammatory, hemorrhagic and potentially necrotizing [8,9]. *E. ocellatus* is responsible for more than 80% of envenomation cases treated in hospitals and almost all of the deaths in the Savannah region.

Viper venoms are responsible for hemorrhagic and necrotizing complications involving many organs, starting with tissue at the bite site. Following envenomation by *Echis ocellatus*, many hemorrhagic complications have been described: stroke and subarachnoid hemorrhage [11,12], hemothorax [13], hemoperitoneum [14,15] and kidney injury [8,16-17]. Well documented in Asia [18-21] and in South America [22,23], snake venom associated-acute kidney injury (AKI) is poorly explored in SSA and has never been studied in Benin, despite an estimated frequency of 15% of envenomations [8]. In view of this scenario, the aim of this prospective study is to describe clinical, biological and ultrasonographic aspects of AKI after severe viper envenomation in Benin.

## Methods

### Study location and population

The survey took place in the intensive care unit of the Parakou University Hospital (PUH) that includes a medical imaging service and a biology laboratory. PUH has 215 beds, including 11 in UCI. Ethical clearance was obtained from Comité local d’éthique pour la recherche médicale of Parakou University on November 13^th^, 2018 (Ref 152/CLERB-UP/P/SP/R/SA). The study was carried out over a period of 18 months (February 1, 2017 to August 31, 2018). The study population consisted in all patients admitted to intensive care for severe envenomation by snakebites and who gave their informed consent. All patients showing symptoms of severe envenomation (inflammatory, hemorrhagic, necrotic and/or neurotoxic syndrome) necessitating resuscitation were included. Patients who died on admission and those with a known history of kidney injury, diabetes or hypertension were not included in the study.

### Physical examination

Informed consent form drawn up in accordance with the ethic committee, was presented and explained to each patient before he/she signed it. A case report form (CRF) was completed during patient’s clinical examination and follow-up. It specified the patient's medical history and results of biologic and ultrasonographic exams.

Demographic data included gender, age, occupation, place of residence and education level. For each patient it was mentioned the type of admission (spontaneous or referred from another health center), place and circumstance of the bite, anatomical site, eventuality of traditional treatment before presentation and general health condition on admission.

Inflammatory syndrome associated pain, edema and, in some case, leukocytosis and fever. Edema was assessed according to the extension relative to involved joints, i.e. grade 0 for absence of edema, grade 1 when only one significant joint (ankle or knee, wrist or elbow) was concerned, grade 2 when edema covered the entire bitten limb and grade 3 when it exceeded the root of the bitten limb.

Hemorrhagic syndrome was established by abnormal whole blood coagulation test (WBCT) and/or externalized or internal bleeding. The WBCT was noted grade 0 when the blood coagulated normally within 20 minutes, grade 1 for partial or crumbly clot, and grade 2 for uncoagulable blood [24]. Clinical bleeding was graded 0 for the absence of visible bleeding, 1 for local bleeding, 2 for inconspicuous but continuous bleeding from mucous membranes (bleeding gums, epistaxis), skin (hematoma, purpura, blisters) or recent scar, and 3 for extensive, multiple and/or internal hemorrhages (digestive, cerebral, meningeal, peritoneal, kidney).

Neurological examination sought pathognomonic symptoms of Elapid envenomation (paralysis of the cranial nerves including ptosis, miosis, respiratory paralysis associated or not with conscience disorders).

Clinical examination of kidney function looked for manifestations of AKI based on urine coloration (normal, dark or black), diuresis (normal, i.e. more than 800 mL urine per 24 hours, oliguria, 100-800 mL or anuria, < 100 mL), proteinuria and hematuria performed by urine test strip (UTS). Diuresis was monitored every 6 hours for the first 24 hours and then, every 12 hours until the patient was discharged.

The following biological data were collected: hemoglobin, blood count (red and white blood cells), platelets, prothrombin time (PT), activated partial thromboplastin time (aPTT), serum creatinine (SCr), serum urea (SUr, blood electrolytes including serum sodium and potassium, 24-hour proteinuria, serum calcium, and serum proteins.

A thoracoabdominal ultrasound was performed on each patient by a radiologist, looking for internal bleeding [14] and kidney damage by visualizing the morphological and structural abnormalities of the kidneys. Kidney ultrasounds were performed with a Mode B brand “Mindray^®^” model “DP 8800 Plus” device and associated probe at variable frequencies (2-5MHz). The ultrasound noted the size of the kidneys (discrete quantitative variable), the normal value of the size of the kidney being between 85 and 120 mm [25] and ultrasound structure of the kidney parenchyma (qualitative variable). AKI was documented by kidney enlargement, subcapsular hematoma, calyx dilation, compressed medulla and/or changes of the corticomedullary aspect.

Treatment consisted, whenever possible, in the administration of antivenom (Inoserp^®^ Panafrica, Inosan Biopharma, Mexico), that benefited from a clinical evaluation [26], and the only antivenom registered in Benin. Batches 6IT05001 and 7IT04004 (mainly the latter) neutralizing more than 500 lethal doses 50% (LD_50_) of *E. ocellatus* venom per vial were used through the study. Patients received analgesic treatment (paracetamol, nefopam, tramadol depending on the intensity of the pain) and tetanus prophylaxis. In addition, blood transfusions (blood cell and/or fresh frozen plasma) and antibiotics (cloxacillin, amoxicillin + clavulanic acid, ceftriaxone or metronidazole) were administered according to observed troubles.

Finally, we collected evolutionary data (length of hospital stays, complications), and outcome (discharge alive or deceased).

The variable of interest was AKI, defined according to the criteria of the Kidney Disease Improving Global Outcomes (KDIGO) 2012 revised in 2017 [27,28]. An AKI was assessed on the basis of SCr increase or urinary output decrease (Table 1), considering that only one of which was sufficient to establish the stage of severity (Table 1). AKI was also documented by kidney ultrasound.

**Table 1:** Classification of acute kidney failure according to KDIGO.

Stage	Serum creatinine	Diuresis
1	Increase 1.5-1.9 times baseline OR Increase ( 3 mg/L^-1^	( 0.5 mL/kg/h > 6 - 12 h
2	Increase 2.0-2.9 times baseline	( 0.5 mL/kg/h > 12 - 24 h
3	Increase 3 times baseline, OR Increase ( 40 mg/ L^-1^, OR Initiation of renal replacement therapy, OR For patients < 18: glomerular filtration rate < 35 mL/min/1,73 m^2^	0.3 mL/kg/h > 24 h, OR Anuria > 12 h

From: "KDIGO Clinical Practice Guideline for Acute Kidney Injury" by Kidney Disease: Improving Global Outcomes (KDIGO), 2012.

*Data analysis*

Data were entered and analyzed using Epi Info^®^ software from the Centers of Disease Control (CDC), versions 3.5.1 and 7.1.1.14 respectively. Quantitative variables were expressed by mean (95% confidence interval) for those showing normal distribution (assessed by visual test), or median [interquartile, IQ: 25-75%] when the distribution was not Gaussian. They were analyzed with parametric (Student's t-test, analysis of variance) and non-parametric (Mann-Whitney or Kruskal-Wallis) tests according to the number of involved (non-parametric for numbers below 25) and distribution of the variables. In case of doubt on the latter, the two types of tests were used in seeking a convergence of the results. Qualitative variables were expressed by proportion with a 95% confidence interval and tested by χ^2^. The significance level was p = 0.05.

## Results

**Figure 1.**
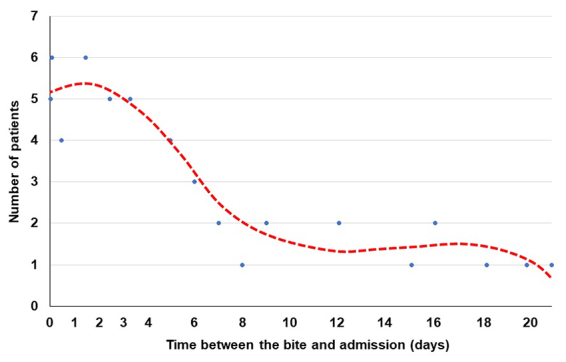
Distribution of time between bite and hospital presentation.

During the study period, 51 patients hospitalized in intensive care of the HUP were included in the study for severe snakebite envenomation. Twenty-one patients (41%) were referred to the UPH from peripheral health facilities lacking therapeutic means. Two patients died within the first 24 hours. One died from sepsis and the other died from acute respiratory distress. None of the patients had history of AKI, diabetes or hypertension. They were not assessed for renal function and not included in the study. Table 2 shows the distribution of patients according to demographic, clinical and biological data at admission. The median time to hospital presentation was 3 days [IQ: 1-7.5; extremes 1-21] (Figure 1).

**Table 2.** Distribution of patients on admission according to clinical and biological variables, comparison between patients with and without AKI

Variables	All patients (n = 51)*	No AKI patients (n = 35)	AKI patients (n = 16)	P
Gender (sex-ratio M/F)	1.55	0.94	7	10^-2^
Age (y)	26 [16; 35.5]	24 [16; 36]	30 [24; 36.8]	0.23
Traditional treatment	43/51 (84%)	30/35 (86%)	13/16 (81%)	0.68
Median time to admission (days)	3 [1; 6.5]	4 [1; 7]	3 [1; 4.5]	0.61
Bleedings (Grade)	46/51 (90%) - Grade 2 [1; 2]	32/35 (91%) - Grade 2 [1; 2]	14/16 (88%) - Grade 2 [2; 2]	0.25
Abnormal WBCT (Grade)	51/51 (100%) - Grade 2 [2; 2]	35/35 (100%) - Grade 2 [2; 2]	16/16 (100%) - Grade 2 [2; 2]	0.51
Edema (Grade)	51/51 (100%) - Grade 3 [2; 3]	35/35 (100%) - Grade 2 [2; 3]	16/16 (100%) - Grade 3 [2; 3]	0.19
Abnormal urine color	20/51 (39%)	11/35 (31%)	9/16 (56%)	0.09
Proteinuria	20/51 (39%)	11/35 (31%)	9/16 (56%)	0.09
Hematuria	5/51 (10%)	2/35 (6%)	3/16 (19%)	0.15
Oliguria	11/51 (22%)	3/35 (9%)	8/16 (50%)	10^-3^
Median hemoglobin (g·L^-1^)	6.2 [4.5; 7.8]	6.2 [4.9; 8.1]	5.6 [4.2; 6.4]	0.24
Median platelet number (x 10^3^)	100 [79; 142]	102 [75; 131]	97 [81.8; 152.5]	0.93
Median leucocytes (x 10^3^)	12.3 [10.8; 14.4]	12.3 [10.8; 14]	13.7 [11; 16.7]	0.26
Median serum creatinine (mg·L^-1^)	13 [11; 60]	12 [9.5; 15]	62 [17; 162.5]	10^-3^
Median urea (g·L^-1^)	0.38 [0.27; 0.44]	0.34 [0.2; 0.4]	0.54 [0.42; 0.87]	10^-3^
Median serum sodium (mmol·L^-1^)	132 [124.8; 137]	132 [122.8; 135]	132.6 (4.3) - 133.5 [126; 138.5]	0.09
Abnormal renal ultrasonography	5/51 (10%)	2/35 (6%)	3/16 (19%)	0.61
KDIGO (stage)	16/51 (31%) - Stage 0 [0; 1]	0	16	-
Antivenom administration	41/51 (80%) - Doses = 2 [1; 3]	28/35 (80%)	13/16 (81%)	0.92
Duration of hospital stay (d)	4 [3; 6]	4 [3; 5.5]	4.5 [2.8; 6]	0.51
Deaths (case fatality rate)	7/51 (14%)	3/35 (9%)	4/16 (25%)	0.11

*n (%); median [IQ 25%; 75%]

### Sociodemographic data

The median age of the patients was 26 years [IQ: 16-35.5; extremes: 7-63] (Table 2). In our series, 31 patients (61%) were male with a sex ratio (M/F) of 1.55. Farmers were the most represented occupational category (n = 20; 39%). The other socio-professional categories identified were: 15 schoolchildren/pupils/students (29%), 7 shepherds (14%), 6 housewives (12%), 2 shopkeepers (4%), 1 civil servant (2%). Twenty-seven patients (53%) were unschooled. Forty-two patients (82%) lived in rural areas.

Thirty-eight patients (75%) were bitten in the bush during an agricultural activity and pastoral for one of them. In contrast, 13 bites (25%) took place at home during domestic activities. Forty-three patients (84%) received traditional treatment before hospital presentation. Medicinal plants, associated with scarifications, black stone or tourniquet, were used in 38 (88%) of the 43 patients.

### Clinical data

Foot was involved in 41 patients (80%), hand in 8 others (16%), chest in 1 victim (2%) and genitals in the last one (2%).

All 51 patients showed abnormal WBCT, median grade = 2 [IQ: 2; 2], and bleeding in 46 of them (90%), median grade = 2 [IQ: 1; 2]. All patients had large edema, median grade = 3 [IQ: 2; 3]. Abnormal urine (color, quantity, proteinuria or hematuria) was observed in 29 patients (57%).

No symptoms suggestive of Elapid bite, including paralysis of cranial nerves (ptosis, dysphonia, respiratory paralysis), was observed in patients of the series. Neurological impairments were compatible with viper envenomation complications.

*Biological data*

As displayed in Table 2, most patients (n = 47; 92%) showed anemia (Hb <13 g·dL^-1^ in men and < 12 g·dL^-1^ in women according to WHO standards), including 38 (75%) severe anemia (<8 g·dL^-1^). There was no difference in the distribution of anemia between male and female. The platelet count was below 150,000 per mm^3^ in half of the patients (n = 25), showing no significant difference according to gender. However, none had a rate below the critical threshold of 30,000 platelets per mm^3^. The median platelet count was 100,000 [IQ: 79,000; 142,000]. Hyperleukocytosis (> 10,000 leukocytes per mm^3^) was present in 34 patients (67%), median 12,100 [IQ: 6,550; 14,350].

Blood electrolyte disturbances were found in almost two-thirds of the 44 patients for whom it was performed, with 27 hyponatremic patients (61%) and only one hyperkalemic (2%). Seven out of 12 patients tested showed hypocalcemia.

Few TP and TCA were performed but they were all abnormal.

Twenty-one patients (41%) had high SCr (> 14 mg·L^-1^) at presentation. The kidney tests performed after 48 hours of hospitalization noted that an additional 5 patients showing high SCr, bringing the total number of patients with high SCr to 26 (51%). Referring to the KDIGO criteria, AKI was confirmed in 16 patients out of which 13 showed stage 1, 1 stage 2 and 2 stage 3.

*Ultrasonographic data*

**Figure 2.**
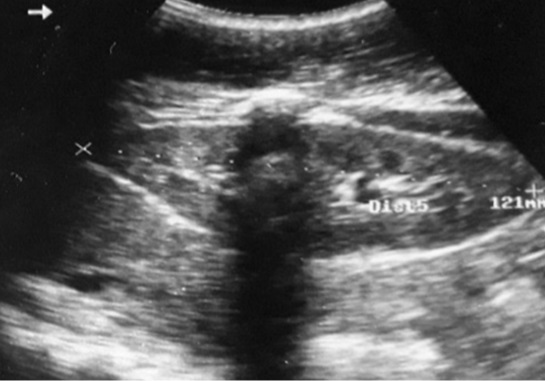
Ultrasound image of enlarged kidney. Right kidney measuring 121 mm in length with good corticospinal differentiation.

**Figure 3.**
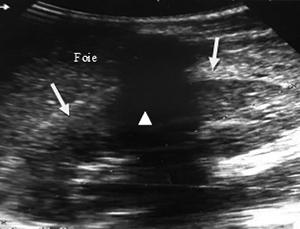
Ultrasound image of a small hematoma under the right kidney capsule. Hyperechoic border of liver and kidney (white arrow), arcuate measuring 11.6 mm thick.

**Figure 4.**
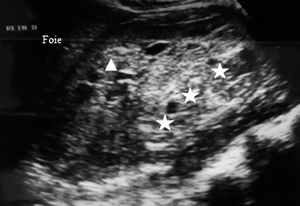
Ultrasound image of a large hematoma under the capsule of the right kidney. Heterogeneous hyperechoic formation (white stars) in median subcapsular position pushing off the right kidney (white triangle).

**Figure 5.**
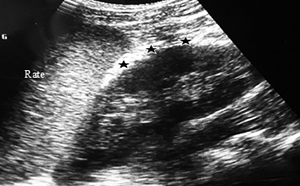
Ultrasound image of a small subcapsular hematoma of the left kidney. Hyperechoic arcuate border (black stars) between the spleen (*rate* in French) and kidney.

**Figure 6.**
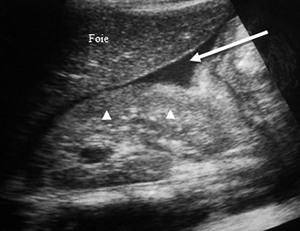
Ultrasound image of a hemoperitoneum in Morrison's lodge and kidney stage 1 injury. Hypoechoic collection (white arrow) in Morrison's lodge between the liver (*foie* in French) and the right kidney (white triangles) of echogenicity equal to the liver (stage 1 kidney disease).

**Figure 7.**
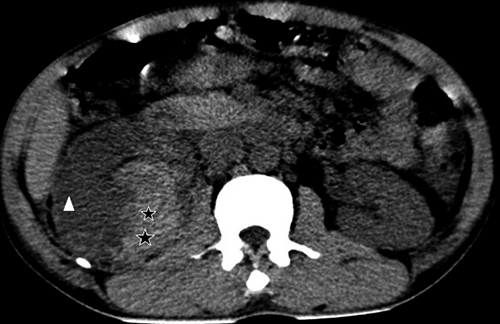
Ultrasound image of a subcapsular hematoma of the right kidney in axial section without injection of contrast medium. Spontaneous hyperdense structure under the right kidney capsule (black star) compressing the right kidney (white triangle).

Ultrasound exploration was normal in 29 patients (57%). Extra kidney abnormalities (hemoperitoneum and hemothorax) were observed in 17 patients (33%). Ultrasound revealed in 8 of 16 AKI patients, 3 cases (19%) of kidney capsular hematoma, 2 cases (13 %) of kidney hypertrophy (Figures 2-7).

*Treatment*

Antivenom was administered to 40 patients within average time of 5 ± 3 hours after admission (range: 1-96 hours). It was renewed in 35 patients (83%). A total of 101 vials were used, i.e. an average of 2.5 vials of antivenom per treated patient. AKI occurred in 13 patients (33%) who received antivenom and in 3 patients (27%) who did not receive it (p = 0.74). 

Five patients showed mild adverse events such as pruritus or papule that were not precisely documented but considered to be potentially attributable to the antivenom. Symptoms disappeared spontaneously or with a dose of oral antihistamine.

One patient underwent hemodialysis. He was admitted 7 days after the bite and benefited from 3 successive daily hemodialysis from the 3rd day after admission and recovered 4 days following the beginning of hemodialysis.

Surgery was performed in two patients to remove the necrotic lesion.

*Evolution and outcome*

The median length of hospital stays was 4 [IQ: 3; 6] days with extremes of 2-11 days. There was no significant difference between the groups without and with AKI.

Complications occurred in 15 patients (29%). During hospitalization, 14 hemoperitoneum, 10 superficial necrosis of the bitten area, 3 afibrinogenemia syndromes and 2 hemothorax occurred.

Seven patients died (14%), including 3 in the group of 35 patients without AKI and 4 in the group of 16 patients with AKI (P> 0.11). None of the 3 patients without AKI received antivenom. In the AKI group, three patients received antivenom. In two of them, dialysis was not performed and they died without kidney function recovering. Only 2 vials were administered to each of the patients.

### Associated factors with AKI

Table 2 compares the set of variables between the two groups of patients, with and without AKI. The univariate analysis showed significant link to gender, diuresis reduction, SCr, SUr and proteinuria (Table 2). We did not consider diuresis and SCr as independent variables since they entered AKI definition according to KDIGO criteria, nor SUr and proteinuria which are directly impacted by AKI. AKI affected 14/31 (45%) males and 2/20 (10%) females (P = 0.008). In our study, gender therefore appeared to be the only variable associated with AKI.

## Discussion

All patients showed inflammatory and blood clotting disorders (i.e. at least abnormal WBCT) on admission. In addition, after carrying out the kidney check-up at presentation and 48 hours, 16 patients presented AKI according to KDIGO criteria [27,28] out of which 13 showed stage 1 (81%) i.e. low severity. AKI resulting from a snakebite therefore seems as common in Benin as in Asia or Latin America [29].

The snake responsible for the bite has not been formally identified. However, the symptomatology, especially intensity of bleedings, leaves little doubt: *E. ocellatus* is probably responsible for most if not all of the bites. *Bitis arietans* also causes coagulation disorders but the bites are much rarer and usually the patients well distinguish between “small” vipers and “large” vipers. However, AKI incidence and severity could be of higher importance after *B. arietans* bites [30]. Finally, spitting cobras (*Naja nigricollis*), cause significant skin necrosis but no coagulation disorders. 

During snakebite envenomation, three mechanisms may cause AKI [31]:

- an immunological reaction (anaphylaxis, action of immune complexes);
- inflammatory syndrome and hemodynamic disorders, directly related to the intensity of the patient's innate defense response and bleeding;
- direct toxic action of the venom on the kidney parenchyma.

The immunological mechanism is likely to play a minor role [32]. Hemodynamic disorders result in peripheral vasodilation, cardiotoxicity and hypovolemia, which are common in snakebite envenomation. Symptoms and evolution are very similar to sepsis that induces decrease in systemic vascular resistance, increase in cardiac output and kidney vascular resistance which lead to a decrease in kidney blood flow and glomerular filtration rate. Many snake venom peptides and enzymes impact blood pressure regulation, peripheral vasomotricity, integrity of vascular endothelium and kidney sodium reabsorption [33-35]. Phospholipases A_2_ (PLA2s) activate the metabolism of prostaglandins and leukotrienes which play a central role in the inflammatory response through the metabolic cascade of arachidonic acid. Angiotensin converting enzyme inhibitors potentiate the effect of endogenous bradykinin. Snake venom serine proteases (SVSPs), which are mainly involved in the blood clotting cascade, activate the kallikrein-kinin system which has an important function in inflammation and controlling blood pressure. Zinc-dependent metalloproteases (SVMPs) hydrolyze basement membrane of vascular endothelium promoting blood leakage, progress of edema and hematoma, and low blood pressure. Natriuretic peptides (SVNPs) inhibit the reabsorption of sodium by the kidney tubules. All these factors are present in the venom of *E. ocellatus* [36] with redundancy which strengthens their power.

In some cases, AKI is not associated with hemodynamic disorders or major inflammatory and hemorrhagic syndromes, suggesting direct nephrotoxicity [37-40]. In numerous studies, mesangiolysis, glomerulonephritis and vasculitis have been observed in snakebite envenomation [41]. It is likely that, in addition to the deleterious effects already mentioned, some enzymes, notably PLA2s and SVMPs which are particularly abundant in the venom of *Echis ocellatus*, damage the nephron. PLA2s hydrolyze the lipid layer of kidney cells and SVMPs break proteins in the extracellular matrix, perforating it, inhibiting cell adhesion, destroying membrane receptors and activating mediators of inflammatory response and apoptosis.

Kidney biopsy has diagnostic, prognostic and therapeutic value for patients who do not show satisfactory improvement of kidney function three weeks after the bite [21]. The main pathological lesions reported by Vikrant et al. [21] in snake venom associated AKI are acute tubular necrosis (ATN) in approximately 80% of patients with confirmed AKI, often associated with interstitial nephritis [42], and much more rarely kidney cortical necrosis (NCR) with poor prognosis, glomerulonephritis or papillary necrosis. Characteristic histological lesions consist in focal hemorrhages and fibrin deposits which reflect a thrombotic microangiopathy leading to NCR [43,44].

Finally, positive correlation with the plasma concentration of venom has been confirmed by several authors [37,45].

AKI incidence in our hospital series is high and falls within the upper limits of the incidence reported by many authors in both Asia and America (Table 3). AKI symptoms and histological lesions are summarized in Table 4. Most snakebites do not progress to serious complications requiring hospital care. Almost half of people bitten by snake do not present symptoms (dry bite) and a large number of envenomed patients do not attend hospital [6].

**Table 3.** Comparison among the main studies on AKI

Country	Time period (years)	Total patients	Total AKI (% total patients)	Sex-ratio AKI patients	Mean time to admission (days)	Bleeding (%)	RRT* (%)	Platelets < 150·10^-3^ (%)	Mean creatinine (mg·L^-1^)^$^	Mean urea (mg·L^-1^) ^$^	Case fatality rate (%)	References
India (North)	22	246	70 (28%)				76%	14%	92 ± 34	233 ±107	29%	[42]
Brazil (South)	3.6	100	29 (29%)	4.8	< 0.5	83%	24%				10%	[48]
Taiwan	2.6		13	5.5	< 0.5		69%	38%	18 ± 5	261 ± 56	0	[61]
India (South)	2	1,548	159 (10%)	1.3	0.9	28%	45%	25%	42 ± ND	100 ± ND	23%	[49]
Turkey (West)	7	200	16 (8%)	1	0.1		25%	60%	29 ± 5	113 ± 78	18%	[46]
India (South)	10	533	143 (27%)	2.3	< 1		57%				21%	[40]
India (Center)	1.6	246	109 (44%)	5.3^&^	> 1.3^&^	23%^&^	52%	42%^&^	66 ± ND^&^	148 ± ND^&^	16%	[55]
India (Center)	1.7	281	87 (31%)	2.3	0.9	83%	55%		49 ± 25	163 ±83	39%	[50]
India (South)	1	246	36 (15%)	1.3	0.9	39%	44%		31 ± 15	85 ± 29	22%	[51]
Brazil (North)	10	276	42 (15%)	3.2	1	52%	31%		30 ± 29	107 ± 74	0	[52]
India (South)	1.6	61	28 (46%)	1.2	0.2	38%	36%				14%	[62]
China (Center)	1.8	119	16 (13%)	1	1.3						6%	[47]
Pakistan	25		115	1.6	8.8	65%	92%				13%	[19]
India (North)	1.5	138	62 (45%)	4.2	2.8	50%						[53]
India (North)	1.3	460	155 (34%)	2.2	3.2	66%	100%	10%	46 ± 2		30%	[63]
India (North)	13	447	121 (27%)	0.7	3.4	87%	82%	48%	72 ± 42	169 ± 75	9%	[21]
India (Center)	1.8		100	1.6	> 0.5	64%	12%	26%	23 ± ND	610 ± ND	6%	[56]
Myanmar	1.3	258	140 (54%)	3.7		60%	49%		22 ± ND	597 ± ND	19%	[20]
Brazil (North)	2	345	24 (7%)	4.4	< 0.5	58%	17%	100%			0	[54]
Brazil (North)	1	63	22 (35%)	0.8	< 0.5		5%					[23]
Benin (Center)^$^	1.6	51	16 (31%)	7	8.5	88%	6%	69%	86 ± 39	60 ± 10	25%	This study

*RRT: renal replacement therapy; ND: not mentioned; $: creatinine and urea are expressed as mean ± CI for comparison with the other studies rather than median [IQ] as used in the paper; &: based on 57 patients with severe AKI.

**Table 4.** Clinical and pathological manifestations of AKI following snakebite envenomation

Clinical symptoms	Pathological changes
Hematuria	Vasculitis
Proteinuria	Thrombotic microangiopathy
Oliguria (+ dark colored urine)	Glomerulonephritis
Nephrotic syndrome	Tubular necrosis
Hemoglobinuria	Interstitial nephritis
Myoglobinuria	Cortical necrosis
Hemolytic uremic syndrome	
Acute kidney failure	
Hepatorenal syndrome	

Modified from: Sitprija and Sitprija [31].

Gender, with a sex ratio of 7 in AKI patients compared to 0.94 in non AKI patients, showed a highly significant difference. Higher incidence of AKI in males has been found by most authors (Table 3), apart from Danis et al. [46] in Turkey, Li et al. [47] in China, Vikrant et al. [21] in India and Albuquerque et al. [23] in Brazil. To our knowledge, this difference has not been explained. One explanation could be that males and females are bitten by distinct snake species. However, it cannot be the only cause since, in our study as in several others, the same species was responsible for bites in both males and females [33,37,45]. In addition, it has been underlined that the incidence of envenomations is always higher in males [6] and, in many studies, similar sex ratio was observed between patients without and with AKI [40,48-54]. Nevertheless, we have shown that the incidence of AKI was very significantly higher in males, independently of the risk of bite also higher than in females. In order to explain sex ratio difference, we failed to find gender associated factors in our series, including the delay in hospital presentation (p = 0.67), age (p = 0.39) and bleeding severity (p = 0.16).

Although AKI patient's median age was higher than those without AKI, the difference in our series was not significant, whereas it was in several studies [21, 45, 47, 49, 52,53]. This could be due to the small size of our series.

Traditional treatment has been reported as a risk factor [51]. Plant nephrotoxicity cannot be excluded, however, this would more likely be a confounding factor with treatment delay, which constitutes a significant risk of AKI in most studies (Table 3). Presentation delay was significantly correlated with the risk of AKI [47,51]. It was not significant in our series, probably because most patients attended hospital very late (Figure 1), well after the critical threshold which allows prevention of AKI by antivenom administration. Presentation delay was explained by the distance between snakebite place and hospital, patient's transportation logistic, treatment seeking behavior favoring traditional medicine. In addition to the time before admission, antivenom administration occurred 5 ± 3 hours on average after hospital presentation. This was the consequence of antivenom inaccessibility, the cost of which (42,000 FCFA or US $ 70 per vial) representing several months of income for a Beninese farmer. The critical threshold of treatment delay was estimated by many authors below 2 hours after snakebite [20,48,49,53]. However, Alves et al. [54] reported that 58% of the AKIs in their series were admitted to hospital less than 3 hours after the bite -meaning that early treatment was unable to prevent AKI-, and Ratnayake et al. [45] did not show any difference according to treatment time between patients without AKI and those with AKI whatever the severity. Actually, without questioning the correlation between the treatment time and AKI incidence, it is likely that the critical threshold depends strongly from the protocol and circumstances, which does not exclude the possibility of developing an AKI, even if the probability is lower after early antivenom.

Most patients showed abnormal hematological parameters. Anemia, hyperleukocytosis and thrombocytopenia were constant although, in our series, the difference between patients without and with AKI was not significant. However, it is likely that the combination of inflammatory and hemorrhagic syndromes impacted kidney function also in some non AKI patients. Clinical hemorrhages, uncoagulable blood, reduced hemoglobin, hyperleukocytosis and/or thrombocytopenia were observed during AKI (Table 3) and were generally significantly higher in AKI patients [20,45,50,52-56].

Among the biological parameters of kidney function, only diuresis, SCr and SUr differed significantly between AKI and non AKI patients. However, these variables were directly or indirectly related to the definition and grading of AKI. On the other hand, other markers of kidney function (urine coloration, proteinuria, hematuria and serum sodium) were not significantly different between the two groups, whereas significantly modified in studies involving more patients [20,45,48,50,54,55]. Hyponatremia is a poorly specific but sensitive indicator of AKI, reflecting inadequate reabsorption of sodium by the renal tubule. Serum sodium below 135 mmol·L^-1^ should lead to kidney function exploration. In addition to the small number of patients in our series which probably explained this lack of significance, Aye et al. [20] showed that the loss of electrolytes, especially serum sodium, was frequent in non AKI patients limiting the relevance of hyponatremia as AKI indicator.

Proteinuria and hematuria generally reflect glomerular lesions resulting from hemodynamic disorders and/or intense inflammatory response [21,23,38]. Inexpensive, easy and rapid to perform, UTS would allow first-line screening for AKI. Considered separately, each of these variables showed poor performance. However, together abnormal color of urine, proteinuria and/or hematuria, increased UTS sensitivity to 69% and specificity to 63%, with a positive predictive value (VPP) and negative predictive value (VPN) of 46% and 81% respectively.

We did not observe chronic kidney injury during the months following the bite, as it has been reported in Asia and Brazil [21,47,52,57].

In few patients, ultrasound revealed subcapsular hematoma, kidney enlargement and kidney damage at different stages, most likely linked to hemorrhagic syndrome. Ultrasound kidney abnormalities following snakebite envenomation have been rarely reported in the literature apart from several studies in South Asia, especially India. Patil et al. [55] reported normal-size kidneys in all patients in their series and ultrasonic structure changes with conserved cortico-sinus differentiation. Golay et al. [58] described spontaneous peri-nephritic hematoma in a patient after snakebite envenomation.

Mortality after snakebite envenomation generally result from hemorrhagic syndrome which leads to severe anemia, hypovolemic shock, or cerebral and subarachnoid hemorrhages. The difference in case fatality rate between patients without and with AKI was not significant (p = 0.11) and there was no relationship between the severity of AKI assessed by KDIGO score, and risk of death (p = 0.86). None of our patients appeared to have died directly from AKI consequences. The parameters showed severe inflammation and bleeding in all deceased patients. However, while moderate, AKI certainly worsened the condition of patients 4-7 and probably partly contributed to the cause of death (Table 5). AKI could also explain shorter time between bite and death compared to non AKI patients, although non-significant (p = 0.11). Otherwise, 4 of 7 deceased patients did not receive antivenom. The considerable delay between the bite and arrival at hospital (> 12 days) made the administration of antivenom irrelevant since the venom disappears from the body in less than 10 days [59].

**Table 5.** Demographic, clinical and biological variables of deceased patients at admission

Parameters	Patient 1	Patient 2	Patient 3	Patient 4	Patient 5	Patient 6	Patient 7
Gender	Male	Male	Male	Male	Male	Female	Male
Age	24	42	41	42	18	32	42
Time to admission (days)	12	16	21	1	3	6	16
Grade of bleeding	2	1	2	2	2	2	2
Grade of WBCT	2	2	2	2	2	2	2
Grade of edema	3	2	3	3	3	3	3
Abnormal urine*	Yes	Yes	Yes	No	Yes	Yes	Yes
Diuresis	Normal	Normal	Normal	Anuria	Oliguria	Normal	Normal
Hemoglobin (g·L^-1^)	4.5	6.4	4.5	3.5	3.4	6.2	4.3
Platelet number	48,000	30,000	72,000	159,000	152,000	101,000	82,000
Leucocyte number	13,200	18,000	11,000	14,300	28,000	11,000	17,200
Serum creatinine (g·L^-1^)	13	12	11	164	195	64	12
Abnormal renal ultrasonography	No	No	No	No	Yes	No	No
KDIGO stage	0	0	0	1	1	1	2
Renal replacement therapy	No	No	No	No	No	No	No
Antivenom administration	No	No	No	Yes	Yes	Yes	No
Duration of hospital stay (days)	3	3	3	2	2	6	2
Time between bite and death	15	19	24	3	5	12	18
Probable death cause	Bleeding, anemia	Bleeding, anemia	Bleeding, anemia	Bleeding, anemia	Bleeding, anemia	Bleeding, anemia	Bleeding, anemia

*Abnormal urine: dark color, and/or proteinuria and/or hematuria.

Treatment of envenomation has two components: on the one hand, antivenom quickly eliminates the venom from the patient and, on the other hand supportive and symptomatic treatments intend to treat venom-induced disorders and patient exaggerate defense response. Clinical and biological troubles are correlated to the venom quantity and toxicity [59-61]. Symptoms can be exacerbated or prolonged, both by the patient's individual response and poor envenomation management. This is often the case in SSA where medical equipment, medicine supply and patient's financial means are insufficient and do not allow rapid and adequate treatment. Finally, incidence and severity of complications are strongly related to the time between the bite and patient care, making it a decisive factor.

Antivenom dose should be determined by the amount of venom injected by the snake. This is not known a priori but can be estimated from patient's symptoms. Antivenom, administered early, quickly controls bleeding and coagulation disorders, which impact significantly the onset and course of AKI [20,21,48,49,52-54,61].

Symptomatic treatment of AKI includes the treatment of coagulation disorders, in particular administration of coagulation factors (blood infusion, fresh frozen plasma, packed red blood cells, as far as they are available), diuresis boost and kidney replacement therapy, either by hemodialysis or peritoneal dialysis according to the possibilities. However, dialysis whatever the type does not always seem to be effective [49, 50, 55]. Diuresis boost requires restoring blood volume, ideally with an isotonic solution (Ringer lactate or saline 9 ‰), electrolyte balance and administration of reasonable doses of diuretics and vasoactive drugs (norepinephrine, dopamine, dobutamine), under careful monitoring diuresis and SCr [52].

The main asset of the study was to be one of the first surveys regarding snakebite AKI in SSA. However, the study showed some limitations. First, it is a small series of patients not representative of all the envenomations observed in SSA. It is therefore not possible to deduce the actual incidence of AKI. Second, almost half of patients first consulted in a peripheral health center (and before that, traditional healer) delaying hospital presentation and, possibly, interfering with the progression of the envenomation. However, although our sample was biased since the PUH is a national reference hospital which concentrates severely envenomed patients, it should be underlined that more than half of the patients in this series were admitted directly without being referred by a peripheral health center.

This study was not intended to measure the severity of AKI, or to determine etiology and pathophysiology of kidney lesions, which would have required kidney biopsies. Our limited means did not allow to fully explore the kidney function and its progression during the months following hospitalization, in particular concerning the possible development towards chronic kidney injury.

Finally, the protocol of this prospective observational study was not intended to validate standardized management, in particular the duration of treatment, the dose of antivenom and symptomatic treatment.

The different issues must therefore be the subject of further studies which will also make it possible to define early markers of AKI according to the severity.

## Conclusion

AKI is a common complication of severe snakebite envenomation. Although it concerns up to 30% of severe viper envenomation showing inflammatory and hemorrhagic syndromes, it remains largely underestimated and unknown in SSA. Although apparently moderate, AKI appeared possibly life-threatening by adding to the already high risk of death from bleeding complications. The risk of progression to chronicity could not be assessed by this study and must be investigated.

Hemodynamic disturbances caused by venom seem to be the most common cause of AKI. Treatment delay, often linked to treatment seeking behavior pushing the patient to consult traditional healer, constitutes a factor of poor outcome emphasized by most authors. This study confirmed that AKI is significantly more frequent and severe in males.

AKI should be routinely searched in envenomed patient showing large inflammatory syndrome and/or bleeding. Dark urine, diuresis reduction, proteinuria and/or hematuria using a UTS can provide rapid and inexpensive screening. Treatment should associate early administration of antivenom, and symptomatic treatment aiming at controlling blood clotting disorders, diuresis boost and, possibly, initiating kidney replacement therapy.

Further studies should make it possible to specify the actual incidence and severity of AKI as well as to standardize management.
